# Protocol for electrophysiological monitoring of carotid endarterectomies

**DOI:** 10.1016/S1674-8301(10)60061-9

**Published:** 2010-11

**Authors:** Hong Liu, Anthony M Di Giorgio, Eric S Williams, William Evans, Michael J Russell

**Affiliations:** a Department of Anesthesiology University of California, Davis Medical Center, Sacramento, CA 95817, USA; b Active Diagnostics, Inc., Davis, CA 95616, USA; c Kaiser Permanente Sacramento Medical Center, Sacramento, CA 95825, USA; d Aaken Laboratories, Inc., Davis, CA 95616, USA

**Keywords:** intraoperative monitoring, somatosensory evoked potentials, electroencephalogram, carotid endarterectomy, carotid

## Abstract

Near zero stroke rates can be achieved in carotid endarterectomy (CEA) surgery with selective shunting and electrophysiological neuromonitoring. though false negative rates as high as 40% have been reported. We sought to determine if improved training for interpretation of the monitoring signals can advance the efficacy of selective shunting with electrophysiological monitoring across multiple centers, and determine if other factors could contribute to the differences in reports. Processed and raw beta band (12.5-30 Hz) electroencephalogram (EEG) and median and tibial nerve somatosensory evoked potentials (SSEP) were monitored in 668 CEA cases at six surgical centers. A decrease in amplitude of 50% or more in any EEG or SSEP channel was the criteria for shunting or initiating a neuroprotective protocol. A reduction of 50% or greater in the beta band of the EEG or amplitude of the SSEP was observed in 150 cases. No patient showed signs of a cerebral infarct after surgery. Selective shunting based on EEG and SSEP monitoring can reduce CEA intraoperative stroke rate to a near zero level if trained personnel adopted standardized protocols. We also found that the rapid administration of a protective stroke protocol by attending anesthesiologists was an important aspect of this success rate.

## INTRODUCTION

Traditionally, carotid endarterectomy (CEA) or the surgical removal of plaque from the carotid artery has involved a significant risk of clamp-induced or embolus-induced stroke. This occurs because the procedure requires that either the flow of blood through the carotid artery be interrupted by clamping or a bypass shunt be placed while the carotid artery is opened and the plaque is removed. Institutions with practiced surgeons who perform high volumes of CEA report intraoperative stroke rates of 3-5%[1]–[5] as measured by postoperative symptoms. This number would be expected to significantly increase if evaluated by postoperative MRI or other methods since infarcts in many parts of the brain are asymptomatic[2]. Intraoperative shunting reduces the risk of stroke due to hypoperfusion, but increases the risk of embolic stroke if plaque, air or debris is released in the vessels during shunt placement. Shunts can also cause carotid dissection resulting in high morbidity[3],[4]. Various methods have been practiced to monitor brain perfusion throughout the surgery, determining when shunting of the artery is necessary (selective shunting). These methods vary in cost and effectiveness, but the most controversial in the literature is electrophysiological monitoring (EM) with some reports suggesting that it is the most effective means of monitoring[5]–[8] and others suggesting that it is ineffective[9],[10]. This report attempts to resolve the issue with multiple surgeons, multiple neurophysiologists, and multiple sites, but a common training program.

The specific method of EM varies greatly. Electroencephalogram (EEG) is the most common modality used for monitoring CEA, but somatosensory evoked potentials (SSEP) are sometimes used in conjunction with EEGs.

EEG measures the spontaneous electrical activity of the brain. By measuring the amplitudes of certain wavelengths relative to patient's baseline, brain perfusion can be inferred from the electrical activity. Compressed spectral array (CSA) is a graphical representation of the amplitudes of the wavelengths of interest, making it easier to interpret and detect changes in the EEG[11]. The beta-band of the EEG is the frequency most sensitive to ischemia and a rapid indicator of changes in brain perfusion and should be seen as an indicator for hypoperfusion[12]. Alpha wavelengths are poor indicators of stroke. Although they often show a rapid decline, they can show an increase in activity or remain stable even after brain tissue is irreparably damaged from ischemia[13]. A wide variety of montages have been reported in the literature, with anywhere from 2 to 8 channels per hemisphere of the brain[5],[14]–[17]. However, the authors' rationale for their specific montage use is rarely addressed.

SSEP measures a triggered response from a stimulus to the brain. Typically, the stimulus is given at the median nerve and the SSEPs are used to monitor CEA. The area of the cortex responsible for median nerve SSEP generation lies within the watershed for the middle cerebral artery (MCA)[14]. This makes the median nerve SSEP a good indicator for stroke related to ischemia within the MCA watershed. SSEP of the tibial nerve is an indicator for stroke within the anterior cerebral artery watershed[18]. A reduction in SSEP amplitude of over 50% is indicative of intraoperative ischemia[9],[20]. SSEP are less prone to suppression from anesthetic agents and interobserver variability than EEG, and they also persist longer than the EEG when neuroprotective agents are given, but they cover relatively limited region of the brain. They are a valuable complement to EEG during CEA.

EM requires a team approach involving coordination between the anesthesiologist, surgeon and EM personnel throughout the procedure. A skilled professional is required to set up and maintain the equipment, troubleshoot any problems and interpret the recordings to ensure that the anesthetic regimen does not interfere with the monitoring. EM is initially more costly when compared to other selective shunting techniques such as awake monitoring or stump pressure analysis, but the benefit is superior. Monitoring begins soon after intubation and ends at extubation.

Among the commonly used methods of CEA monitoring, EM has the greatest range of reported success rates, but it is also the most controversial. Schneider *et al.*[14], Ballotta *et al.*[21], Facco *et al.*[22], Whittemore *et al.*[23] and Harada *et al.*[24] all found operative stroke rates less than or equal to 0.8% using selective shunting with EEG. Woodworth[5] found patients with selective shunting under EEG were seven times less likely to have a perioperative stroke when compared to patients with routine shunting. Stejskal *et al.*[25] found SSEP to have a 0.4% false negative rate. Hans *et al.*[9] found EEG to have a false negative rate of 40.6%. McCarthy *et al.*[10] found EEG to have 50% sensitivity and 76% specificity. These data reflect the conflict in the literature over the efficacy of EM. If the results of Schneider *et al.*, Ballotta *et al.*, Facco *et al.*, Whittemore *et al.*, Harada *et al.*, Woodworth *et al.* and Stejskal *et al.* can be applied broadly, EM appears to be very effective at preventing intraoperative strokes. However, given the conflicting data presented by Hans and McCarthy, the results seem to be highly variable. This study attempts to determine if standardizing the monitoring protocol and training can make EM effective at multiple hospitals.

## METHODS

Five-hundred-ninety one patients underwent CEA procedures. Several had repeated surgeries so that the total number of procedures was 668. The CEAs were performed at six hospitals in North America (Northern California and Nevada) areas from 2003-2009. Ten different surgeons performed the CEAs. Sixteen different neurophysiologists performed the monitoring. All neurophysiologists had received a standardized training protocol (Active Diagnostics, USA). The training included: (1) two w of instruction on general intraoperative monitoring procedures; (2) demonstration and training on electrode placement; (3) demonstrations of changes in SSEPs; (4) demonstration and training on EEG analysis with emphasis on the beta band. Trainees were specifically instructed to ignore the larger and more predominant alpha waves because they can be present in individuals with extensive brain damage.

CEA was performed under general anesthesia. Muscle relaxant was given for induction and intubation only. The anesthetic regimen used included no more than 1 MAC of halogenated agent (sevoflurane, isoflurane or desflurane), no more than 50% N_2_O and after intubation no boluses of narcotic or propofol. In addition, 5,000 U of heparin was given three min prior to the cross-clamping of the internal carotid artery (ICA) and the systolic blood pressure was maintained between 140-160 mmHg throughout the entire cross-clamping period.

Subdermal needle EEG electrodes were placed after the patient was intubated. EEG was recorded using the following channels, notated using the 10-20 system of electrode placement: F3-C3, F7-T3, T3-T5, F4-C4, F8-T4, and T4-T6. SSEP recording was obtained using the following channels: Cz′-Fpz, C3′-Fpz, C4′-Fpz, C3′-C4′. SSEP stimulation electrodes were placed over the median nerves at the wrist and the posterior tibial nerves at the ankle. Electrodes were connected to the differential amplifier of the Cadwell Cascade (Cadwell Laboratories, Kennewick, WA). EEG channels were band-passed from 1-70 Hz with an analog-to-digital (A-D) gain of 10 µV/div. SSEP channels were band-passed from 30-750 Hz with an A-D and a gain of 20 µV/div. SSEP signals were recorded using a digital averager. Averages consisted of 100 recordings for the median nerve signals and 200 recordings for the tibial nerve signals. Interleaved SSEP stimuli were given at a rate of 2.11 Hz for each nerve with a pulse width of 250 µs. Stimulus intensities were from 15 to 60 mA as per the neurophysiologist's discretion, using a constant current stimulator (ES5, Cadwell Laboratories). Raw EEG was displayed and Compressed Spectral Array (CSA) was used to analyze raw EEG signals as indicated by the software manufacturer (Cadwell Laboratories). CSA was set to deconstruct the beta band of EEG, from 12.5 to 30 Hz. The display gain for the SSEP and EEG windows, as well as the CSA scale, was left to the discretion of the neurophysiologist. Gain for the SSEP display was typically between 0.8 and 1.6 µV/div. Gain for the EEG window was typically between 10 and 30 µV/div. CSA scale was typically between 100 and 400.

All EEG and SSEP recordings were stored by the software throughout the case. The neurophysiologist also periodically recorded the patient's anesthetic levels, blood pressure and temperature, noting any substantial changes or unusual events.

During the introduction of the program the anesthesiologists were asked if they had a “stroke protocol” that they would initiate when an incident was detected and if they would keep the patient's body temperature at about 35°C during the procedure. Prior to the program some indicated that they did not have a protocol and they were then asked to develop one and have the drugs necessary to implement it when an incident was detected. The stroke protocol generally involved increasing neuroperfusion by increasing blood pressure while providing neuroprotection (high doses of propofol or barbiturate) and maintaining moderately low body temperature. When an incident was detected, monitoring was considered secondary to neuroprotection and large doses of drug were given as needed.

**Fig. 1:**
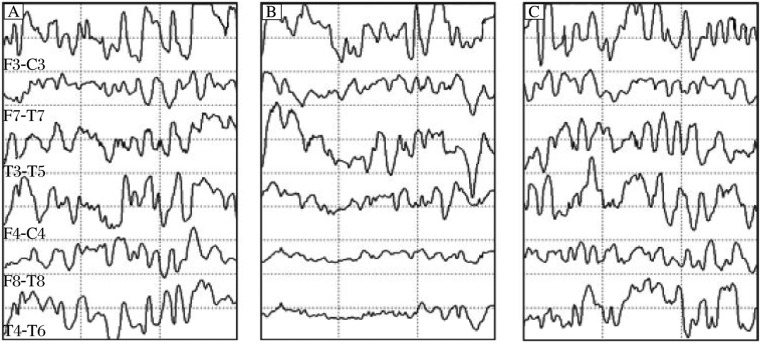
Raw carotid endarterectomy. A: before an ischemic event. B: during an ischemic event. C: during recovery from an ischemic event.

**Fig 2:**
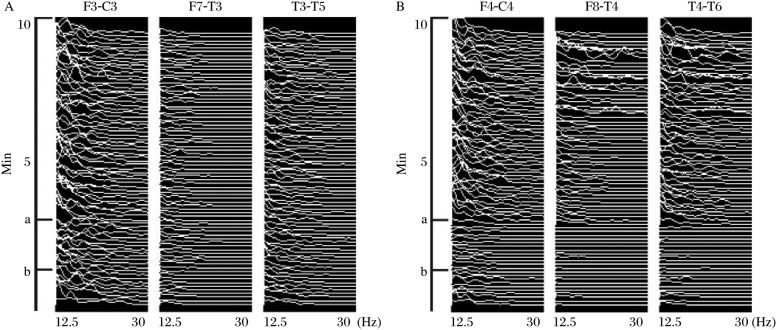
Ten min of compressed spectral array of beta activity. A: compressed spectral array of F3-C3,F7-T7 and T3-T5. B: compressed spectral array of F4-C4, F8-T4 and T4-T6. a: the start of an ischemic event. b: the start of recovery.

**Fig 3:**
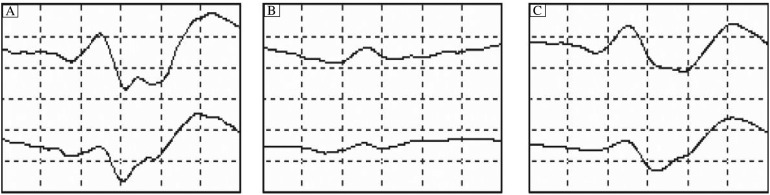
Cortical somatosensory evoked potentials. A: before an ischemic event. B: during an ischemic event. C: during recovery from an ischemic event.

An incident is defined here as a reduction in the power of the beta band (13-30 Hz) of the EEG (***Fig.1*** and ***Fig. 2***) or the amplitude of an SSEP by 50% or more (***Fig. 3***). All incidents were reported to the surgeon and anesthesiologist and noted by the neurophysiologist. For the purpose of this study, incidents are not counted if, according to the judgment of the neurophysiologist, they are the result of increased levels of anesthetic agents. This criterion included: unilateral versus bilateral change and if the change coincided with an increase in anesthetic agents.

## RESULTS

No cerebral deficits were observed as a result of surgery. Incidents that included a reported reduction in amplitude of 50% or more in one of the electrophysiological monitoring modalities occurred in 150 out of 688 cases (22.0%). Of those incidents, 123 occurred during the cross clamp period (82.0% of total incidents). Seven incidents occurred during exposure (4.7% of incidents). Nine incidents occurred during wound closure (6.0%). One incident occurred during patient positioning (0.6%). These numbers add up to greater than 150 because of multiple incidents occurring within a single case. Ten incidents were the result of a reduction in blood pressure and resolved by raising blood pressure and without the use of a shunt. One-hundred-eleven of the 120 incidents (92%) that occurred during cross clamping completely resolved with the use of a shunt. One-hundred-eleven of the 123 (90%) incidents that occurred during the cross clamping occurred within the five min after the ICA was clamped. One-hundred-twenty patients were shunted (***Table 1***).

Nine neurophysiologists monitored at least 25 cases after training. Those nine neurophysiologists accounted for 87% of all the cases monitored. Of those nine neurophysiologists, incident rates ranged between 11.5% and 31.4%. The individual who reported a 31.4% incident rate was returned for additional training. Six surgeons performed at least 50 CEAs, accounting for 97% of all CEAs. Of those surgeons, incident rates ranged between 21.1% and 26.2%.

**Table 1:** Analysis of incidents

Summary of data	
Total number of incidents	150
Incidents with cross clamping	123 (82.0%)
Incidents during exposure	7 (4.7%)
Incidents during closure	9 (6.0%)
Incidents during positioning	1 (0.6%)
Incidents from low blood pressure	10 (15.0%)
Occurred within 5 minutes of clamp	111(74.0%)
Incidents shunted	120 (80.0%)

[*n* (%)]

One patient had gross motor impairment immediately after extubation, but this was later found to be the result of residual paralytic agent that had been administered by the anesthesiologist during the cross-clamping period. Once the paralytic wore off, the patient showed complete muscle strength from all limbs. All other patients demonstrated complete muscle strength from all limbs immediately following extubation. There were no intraoperative strokes or death.

## DISCUSSION

The consistency across six sites and ten surgeons suggest that in experienced hands, these procedures can be highly effective. The low incident rates seen with EM are reproducible and EM can improve morbidity and mortality rates significantly. The complete absence of stroke is consistent with previous research[17]–[20] that shows the superior clinical efficacy of selective shunting with EM while adhering to the above protocol and employing the use of an experienced neurophysiologist. There were no false negative incidents suggesting that with proper training a very high success rate can be achieved. False positive rates are more difficult to determine, because many incidents that would result in transient ischemia do not progress to strokes but would be detected in the electrophysiological record. It is likely that some of the events recorded are false positive, however.

While the focus of this study was the training of the electrophysiologists, it became clear that changes in the anesthesiologist's procedures also played an important role in these very high success rates. The protocol was initiated in the operating room at the onset of an incident and appears to be as important as the selective shunting practiced by the surgeons. The high doses of drug (usually barbiturate or propofol) will reduce or even eliminate EEG and SSEP signals, but the monitoring had preformed its function by issuing an alert and the monitoring became secondary to neuroprotection. Selective shunting has been shown to reduce[5],[14],[24], but not eliminate emboli. Indeed, the placement of the shunt is a significant source of emboli and some must have been released during the study procedure, but the result of this study and those of others have shown near zero rates of stroke. The anesthesiologist plays a significant role with the rapid administration of a neuroprotection protocol. Without the monitoring, an ischemic event would not be recognized immediately and neuroprotective measures may not be applied until the patient awoke, which can be one or two hours after an event or until the patient is transferred to recovery when protective measures would be less effective. This delay greatly increases the risk of ischemic damage to the brain. Neuroprotective measures applied at the time of the event are highly effective and are a likely explanation of the success rates that we and others observed.

Other methods commonly used to monitor during CEAs are carotid stump pressure analysis[28], awake-patient analysis[9], and transcranial Doppler sonography[29]. An extensive literature has been developed with a number of authors advocating the advantages of each of these practices, but none have achieved the success rates observed with EM and selective shunting. There is, however, general consensus among these authors that monitoring and selective shunting presents lower stroke rates than both routine shunting and routine non-shunting[5],[11]–[16].

While the focus of this study was the training and application of a standard protocol by the neurophysiologist, many factors other than the choice of monitoring also affect incident rates. Institutions that perform CEAs regularly have lower incident rates. Quality surgeons are clearly critical, but the factor that seemed to be determinate in this study was the application of a stroke protocol by the anesthesiologist[29]–[33]. When the neurophysiologist alerts the operating room staff that an incident is occurring the surgeon may choose to place a shunt[32],[33], but many of the events occur independently of shunt placement and the shunt itself may cause the release of emboli. When emboli are released, it is the anesthesiologist who takes neuroprotective measures and ensures that cerebral perfusion is adequate.

The data show that, while most incidents occurred around clamping or shunt placement, they also occurred during exposure and closing, suggesting that the monitoring must be performed throughout the case. Individual variances in the skills and methods of the neurophysiologist appear to account for the wide range of incident rates. For instance, in a study comparing EEG to stump pressure and awake analysis, the EM performed by Hans *et al.*[9] did not employ the use of SSEPs and judged a reduction in EEG by a decrease in the power of the alpha wavelength. Using SSEPs greatly improves the ability of EM to detect ischemic events and the alpha wavelength should be largely ignored[13]. The methods used in the current study employed both median nerve and tibial nerve SSEPs as well as CSA of the beta bands.

This study also has some limitations as it does not include follow-up data for the patients that underwent CEA. This study and none of those we have cited have used MRI images of the brains of the patients to determine objective stroke rates. While the costs of such a study would be substantial, a follow-up study that provided long-term stroke free survival rates would be valuable. However, long-term survival with EEG has been studied and the outcome appears equally favorable[21]. Because of the wide range of techniques used in EM, we suggest that a standardized protocol for monitoring CEA and standards in training be developed. This protocol should include both EEG and SSEP and ensure that, while interpreting EEG, beta wavelengths are the focus. Trained and certified neurophysiologists should be employing this protocol for CEA monitoring to ensure that proper EM techniques are being used.

To summarize, the data supports EM as a means of preventing intraoperative stroke during surgery. Different neurophysiologists were placed at different institutions and with different surgeons. When the monitoring was applied with a standardized protocol and coordinated with selective shunting and an integrated stroke protocol near zero rates stroke rates were achieved. While most of the events were associated with clamping, events occurred during all phases of the surgery. Fifteen percent of the events were associated with low blood pressure.
